# Oleanolic Acid Alters Multiple Cell Signaling Pathways: Implication in Cancer Prevention and Therapy

**DOI:** 10.3390/ijms18030643

**Published:** 2017-03-16

**Authors:** Lovro Žiberna, Dunja Šamec, Andrei Mocan, Seyed Fazel Nabavi, Anupam Bishayee, Ammad Ahmad Farooqi, Antoni Sureda, Seyed Mohammad Nabavi

**Affiliations:** 1 Institute of Pharmacology and Experimental Toxicology, Faculty of Medicine, University of Ljubljana, SI-1000 Ljubljana, Slovenia; lovro.ziberna@mf.uni-lj.si; 2 Department of Molecular Biology, Institute Ruđer Bošković, Bijenička c. 54, 10000 Zagreb, Croatia; dsamec@irb.hr; 3 Department of Pharmaceutical Botany, Faculty of Pharmacy, University of Medicine and Pharmacy “Iuliu Hațieganu”, Ghe. Marinescu 23, 400337 Cluj-Napoca, Romania; mocan.andrei@umfcluj.ro; 4 ICHAT and Institute for Life Sciences, University of Agricultural Sciences and Veterinary Medicine, Calea Mănăştur 3-5, 400372 Cluj-Napoca, Romania; 5 Applied Biotechnology Research Center, Baqiyatallah University of Medical Sciences, Tehran 14359-16471, Iran; Nabavisf@gmail.com; 6 Department of Pharmaceutical Sciences, College of Pharmacy, Larkin Health Sciences Institute, 18301 N. Miami Avenue, Miami, FL 33169, USA; 7 Laboratory for Translational Oncology and Personalized Medicine, Rashid Latif Medical College, Lahore 54000, Pakistan; ammadfarooqi@rlmclahore.com; 8 Research Group on Community Nutrition and Oxidative Stress and CIBEROBN (Physiopathology of Obesity and Nutrition), University of Balearic Islands, E-07122 Palma de Mallorca, Balearic Islands, Spain; tosugo@hotmail.com

**Keywords:** oleanolic acid, olive, anticancer effect, signaling pathways, healthy diet

## Abstract

Nowadays, much attention has been paid to diet and dietary supplements as a cost-effective therapeutic strategy for prevention and treatment of a myriad of chronic and degenerative diseases. Rapidly accumulating scientific evidence achieved through high-throughput technologies has greatly expanded the understanding about the multifaceted nature of cancer. Increasingly, it is being realized that deregulation of spatio-temporally controlled intracellular signaling cascades plays a contributory role in the onset and progression of cancer. Therefore, targeting regulators of oncogenic signaling cascades is essential to prevent and treat cancer. A plethora of preclinical and epidemiological evidences showed promising role of phytochemicals against several types of cancer. Oleanolic acid, a common pentacyclic triterpenoid, is mainly found in olive oil, as well as several plant species. It is a potent inhibitor of cellular inflammatory process and a well-known inducer of phase 2 xenobiotic biotransformation enzymes. Main molecular mechanisms underlying anticancer effects of oleanolic acid are mediated by caspases, 5′ adenosine monophosphate-activated protein kinase, extracellular signal–regulated kinase 1/2, matrix metalloproteinases, pro-apoptotic Bax and bid, phosphatidylinositide 3-kinase/Akt1/mechanistic target of rapamycin, reactive oxygen species/apoptosis signal-regulating kinase 1/p38 mitogen-activated protein kinase, nuclear factor-κB, cluster of differentiation 1, CKD4, s6k, signal transducer and activator of transcription 3, as well as aforementioned signaling pathways . In this work, we critically review the scientific literature on the molecular targets of oleanolic acid implicated in the prevention and treatment of several types of cancer. We also discuss chemical aspects, natural sources, bioavailability, and safety of this bioactive phytochemical.

## 1. Introduction

Cancer is known as one of the major global health problems throughout the world, mainly due to changes in lifestyle, as well as unhealthy diets and environmental pollution [1]. Currently, the global map of cancer prevalence is rapidly changing. Referring to Western societies, there has been a decrease in most cancer-related mortality. Meanwhile incidence and mortality continue to rise quickly in developing or undeveloped countries [2,3].

Nowadays, cancer has been considered as an important cause of death and it is estimated that it will be more than 18 billion in the next five years [4]. Despite the substantial progress made in diagnosis and treatment, current therapeutic protocols, such as radiotherapy, phototherapy, chemotherapy, immunotherapy, as well as surgical protocols, are limited and still associated with low outcome, and high morbidity and mortality rate, suggesting crucial needs for finding new effective therapeutic agents [4,5].

The term “chemoprevention” refers to use of pharmacological agents, nutraceutical, as well as bioactive natural products, to prevent or delay cancer development [5]. Recently, much attention has been paid to the clinical usage of natural products as anticancer agents to prevent cancer or delay its development [1,6,7,8].

Epidemiological evidence shows that there is a reverse correlation between high intake of vegetables and fruits and developing non-communicable disease, such as certain types of cancer, stroke, and neurodegenerative diseases [7,9,10]. This statement is particularly ascribed to the presence of a variety of non-nutritive phytochemical agents from plant-based foods [11]. At the same time, a compelling trend and one of the current dilemmas in natural products research is what is more beneficial: the plant extract/herbal product or its active phytoconstituents? The researchers that are in favor of the “whole” argue that the therapeutic efficacy of an extract is the outcome of synergistic or additive effects of its various bioactive components, while those who prefer purified compounds contend that, unlike isolated compounds, several phytochemicals present as a mixture in a natural source are not bioavailable, or have limited bioavailability and, therefore, are less useful. Both arguments present valid hypotheses and, despite the fact that a significant amount of investigation has been carried out in the field, results do not proclaim a winning side [2,11].

When considering the influence of dietary patterns, many efforts have been carried out to evaluate the association between certain dietary models and their health claims as well as the influence of particular single components from the wide range of other nutrients or non-nutrients. Nonetheless, it is worth mentioning that, among various dietary patterns, a traditional Mediterranean diet has been revealed as one of the healthiest choices and this claim is being ascribed to the consumption of copious amounts of olive oil [12]. Despite being less investigated than phenolic compounds, current studies on olive oil triterpenoids, such as oleanolic acid, have revealed a marked potential in altering different cell signaling pathways and, thus, reveal significant potential in cancer prevention and therapy [12,13,14,15,16].

This review presents the chemistry, sources, and bioavailability, as well as the anticancer effects of oleanolic acid, with a particular emphasis on molecular mechanisms of action.

## 2. Chemistry and Biosynthesis

Oleanolic acid (3β-hydroxyolean-12-en-28-oic acid) is one of the most common pentacyclic triterpenoid compounds mainly found in different herbal sources [17]. It is a non-volatile light yellow compound soluble in 1-butanol and ethyl acetate and less soluble in ethanol, 2-propanol, methanol, acetone, and water, where solubility increases with an increase in temperature [18,19]. Triterpenoid compounds possess great pharmacological potential, and their biosynthetic pathways in plants have been thoroughly studied. As reviewed by Pollier and Goossens [17], oleanolic acid biosynthesis goes from the primary sterol metabolism precursor 2,3-oxidosqualene (synthesized in the mevalonate pathway), which is cyclized to β-amyrin by the enzyme β-amyrin synthase. Further, β-amyrin and erythrodiol undergo three consecutive oxidation steps at the C-28 position by the cytochrome P450 (CYP) enzyme to produce oleanolic acid. Oleanolic acid in plants occurs both in its free form, and also as a triterpenoid saponin aglycone linked to one or more sugar moieties. In its free form, it is found in plants cuticular waxes, where it is involved in plant biotic and abiotic stress responses [17].

**Figure 1:**
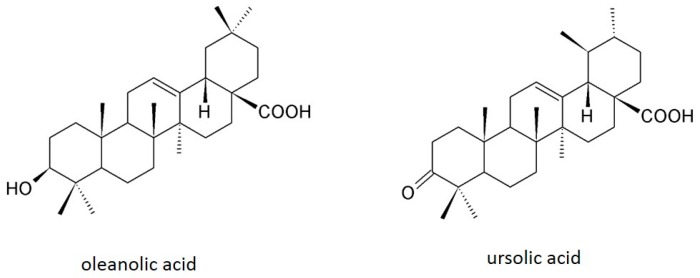
Chemical structures of oleanolic acid and ursolic acid.

In plants and plant-based foods, oleanolic acid is often found together with isomer ursolic acid, from which it differs by the sites of the methyl group on the E loop (Figure 1). Both isomers have similar pharmacological properties [20,21], although they can differ in the intensity of biological activity due to the difference in the position of the methyl groups, which influences their potency and consequently bioactivity of compounds [22]. Oleanolic acid is a registered drug for treating liver diseases in China [23]. In the last 20 years, numerous investigations have been centered on the chemical modifications of oleanolic acid in order to make it more effective and/or develop water soluble derivatives, which resulted in hundreds of new compounds with diverse biological activities. Among the most studied compounds, bardoxolone methyl has been evaluated in several clinical trials for diabetes mellitus, chronic kidney disease, and various types of cancer [16,24].

## 3. Sources and Bioavailability

Oleanolic acid is found in more than 1600 plant species [16]. Aerial parts of higher plants are covered with a hydrophobic layer called the cuticule, which forms a protective coating important for plant survival in its environment. The cuticle contains two structural ingredients, including a polymeric matrix called cutin, and epicuticular wax which can be distinguished in intracuticular and epicuticular layers [25]. Numerous research reported that triterpenoids in plants are found concentrated in the intracuticular wax compartment (reviewed in [25]). Therefore, oleanolic acid content is much higher in fruit “skin” or “peel” (non-botanical terms which include cuticle and multiple cell types) in comparison with the pulp (Table 1). In general, fruits which are consumed with the skin, including dried fruits, can be better dietary sources of oleanolic acid [26]. The most important sources of oleanolic acid in human diet are olives (*Olea europaea* L.), from which the compound derives its name, and their products, such as olive oil [27]. It is estimated that in the Mediterranean diet, containing olives and olive oils, total daily intake of oleanolic acid is around 25 mg [28]. In addition to olives, other foods common in the Mediterranean diet, such as various legumes, contain oleanolic acid in the range of 0.251–2.591 µg/g fresh weight (fw) [29]. High amounts of oleanolic acid are also present in edible parts of jujube (*Ziziphus jujube* Mill.), a commonly consumed fruit in Southern Asia and China [30].

Although triterpenes in free form are found in cuticular waxes of edible plants, roots of medicinal plants, such as ginseng (*Panex* sp.) [31] and wild sage (*Lantana camara* L.) [32], are also high in oleanolic acid. Hawthorn berries (*Crataegus* sp.) [33], and fruits of Chinese privet (*Ligustrum lucidum* W.T. Aiton) [34] and Chinese quince (*Chaenomeles sinensis* (Thouin) Koehne) [35] stand out among other plants used in traditional medicine as a good source of oleanolic acid. Additionally, numerous other medicinal and aromatic plants contain oleanolic acid. Large scale studies have shown its presence in 88 taxa of *Lamiaceae* family [36]. Comparative analysis of 38 commercial herbal extracts showed the presence of oleanolic acid in extracts of *Aesculus hyppocastanum* L., *Crataegus monogyna* L., *Harpagophytum procumbens* DC, *Lagerstroemia speciosa* L., *Ortosiphon stamineus* L., *Punica granatum* L., *Styrax benzoin* Dryand., *Vaccinium myrtillus* L. and *Vitis vinifera* L. [37]. Oleanolic acid is also detected in propolis [37] and numerous plant-based products, including spices [38,39], vegetable oil [27], and snack products, containing dried whole fruits [26].

Biological effects of food components do not depend solely on their amount in certain food, but rather on their bioavailability. Bioavailability is the proportion of a compound that enters the systemic circulation when introduced into the body. Experimental and clinical studies reported the presence of oleanolic acid in blood several hours after intake in its intact form [39,40,41], but oral bioavailability was low due to low aqueous solubility and intensive metabolism by cytochrome P450 isoenzymes (CYP) [42]. Jeong et al. [43] reported that the bioavailability of oleanolic acid after oral administration was only 0.7% in rats. Therefore, in recent years much scientific efforts have been put on creation of complexes and formulations that could increase oleanolic acid bioavailability through increased solubility and permeability [42]. On the other hand, bioavailability does not depend only on physical and chemical properties of a compound, but on the fact that other micro-and macronutrients present in consumed foods affect the bioavailability and metabolism of the targeted compound. In this area, studies on oleanolic acid are still insufficient.

## 4. Oleanolic Acid as Anticancer Agent

### 4.1. Oleanolic Acid Inhibited Tumor Initiation and Development

The anticancer effects of oleanolic acid have been evaluated in many cancer types, including liver cancer [49,50,51,52], lung cancer [53,54], breast cancer [55], colon cancer [56,57,58], bladder cancer [59], prostate cancer [60], pancreatic cancer [61,62], gastric cancer [63], gallbladder cancer [64], osteosarcoma [65], hematological malignancies, e.g., leukemia [66], as well as in central nervous system cancers, such as malignant glioma [67,68].

Anticancer activity of oleanolic acid was initially described by inhibition of the tumor promotion of mouse skin cancer cells under in vivo conditions [69]. In this model, oleanolic acid inhibited chemically-induced carcinogenesis through inhibition of irregular gene expression of several studied genes in mouse skin cancer cells [70]. In another study, chronic oral administration of oleanolic acid (five times a week for four weeks) decreased colon carcinogenesis in rats, showing that oleanolic acid can inhibit tumor initiation [56].

Oleanolic acid dose-dependently suppressed tumor promotion and caused cell cycle arrest at G0/G1 phase in prostate cancer cells [60], at G0/G1 phase in gallbladder cancer cells [64], at S phase and/or G2/M phase in pancreatic cancer cells [62], at subG1 phase in hepatocellular carcinoma cells [71], and at G2/M phase via inhibition of cyclin B1/cdc2 mediated by p21 in hepatocellular carcinoma cells [51,52]. Oleanolic acid treatment of lung cancer cells can also cause cell cycle arrest by modulating miR-122/cyclin G1/MEF2D axis [54]. Modification of the miR-122 activity is a novel anticancer strategy, since this microRNA suppresses cancer cell survival, proliferation, and invasion [72].

Oleanolic acid and its derivatives act as inhibitors of the phosphatidylinositide 3-kinase/protein kinase B/mammalian target of rapamycin/nuclear factor-κB (PI3K/Akt/mTOR/NF-κB) signaling pathway in a dose-dependent fashion [50,55,60]. Another study showed that oleanolic acid inhibited Akt/mTOR/S6K signaling pathway [59]. Oleanolic acid reduced the phosphorylation of PI3K and Akt, which are the upstream molecules of mTOR pathway, whereas there were no significant changes in the expression levels of total PI3K or Akt. These effects are probably mediated by reactive oxygen species (ROS) because treatment of cells with antioxidants, such as *N*-acetylcysteine, reversed the inhibitory activity of oleanolic acid [50]. In addition, over-expression of Akt also reversed the effects of oleanolic acid [60]. Importantly, oleanolic acid is also an inhibitor of extracellular signal–regulated kinase/c-Jun N-terminal kinase/mitogen-activated protein kinase (ERK/JNK/p38 MAPK) signaling pathway mediated by the activation of the 5′ adenosine monophosphate-activated protein kinase (AMPK) signaling pathway that consequently activates downstream signaling leading to the mTOR inhibition and activation of autophagy [55,68,73]. A significant decrease in p-p38α/p38α ratio and in the levels of p-JNK1 and pERK1/2 together with the increased phosphorylation of AMPK were reported in oleanolic acid-treated cancer cells [74]. Oleanolic acid via an AMPK activation-dependent manner also induced metabolic alterations in cancer cells, such as suppressed lipogenesis, protein synthesis, and aerobic glycolysis, thus having tumor suppressor activity [74]. Moreover, it has been demonstrated that ERK activation plays a pivotal role in cancer cells resistant to oleanolic acid’s pro-apoptotic activity [75]. Therefore, pharmacological ERK suppression increased anticancer activity by sensitizing cancer cells to oleanolic acid [75].

From the tumor cell proliferation perspective, oleanolic acid acted as an inhibitor of transforming growth factor-β (TGF-β) by binding to its receptors [76]. Cancer cells respond to excessive production of TGF-β in a pro-tumorigenic manner [77]. In addition, the isomer of oleanolic acid, ursolic acid, inhibited TGF-β/Smad signaling pathways with the antagonistic activity in the low micromolar range (*IC*_50_ = 6.9 µM) [78]. Moreover, oleanolic acid also inhibited topoisomerase I and IIα proteins, which are key enzymes involved in tumor cell proliferation, by relaxing DNA supercoiling inside cells [63]. It has been demonstrated that topoisomerase inhibition activates the NF‑κB pathway [79]. The suppression of Top-I and Top-IIα resulted in the inhibition of the NF‑κB pathway via p‑IκBα and p‑p65‑dependent manner [63].

Oleanolic acid also induced apoptosis through a mitochondrial-dependent pathway by altering mitochondrial membrane potential, releasing caspase activators, such as cytochrome c, into the cytoplasm, leading to the fragmentation of nuclear DNA in human cancer cells [51,71]. The effects can be mediated by the alteration of expression levels of the pro- and anti-apoptotic Bcl-2 families, as evidenced by the decreased expression of anti-apoptotic Bcl-2 and the increased expression of proapoptotic Bax [51].

Although most studies are focused on isolated oleanolic acid and several derivatives, studies on plant extracts containing triterpenes, including oleanolic acid, are also reported. For example, various extracts of *Oldenlandia diffusa*, member of the Rubiaceae family, have been shown to be effective against a number of cancer models, including breast cancer, small cell lung carcinoma, or leukemia cells [80,81,82]. *Crataegus pinnatifida*, belonging to the Rosaceae family, is another example of a medicinal plant with potential effects against cancer cells. Triterpenoids-enriched fraction demonstrated notable antiproliferative activities against liver and breast carcinoma cells [83]. Ethyl acetate extract from *Betula utilis*, a member of the Betulaceae family, exerted cytotoxic activity against cancer cell lines, such as breast, head and neck, lung, ovary, colon, and cervical carcinoma cells [84].

### 4.2. Oleanolic Acid Induced Apoptosis

Oleanolic acid induced apoptosis of tumor cells in numerous cancer cell lines, including acute myeloid leukemia [85], liver cancer cells [50,51], osteosarcoma cells [65], non-small cell lung cancer cells (NSCLC) [53], breast cancer cells [55,86], gastric cancer cells [62,87], pancreatic cancer cells [63], prostate cancer cells [61], bladder cancer cells [59], and colorectal cancer cells [58].

Oleanolic acid and its derivatives induced both extrinsic and intrinsic apoptosis by multiple signaling pathways. Extrinsic apoptosis was induced in human lung cancer cells by oleanolic acid derivative, methyl-2-cyano-3,12-dioxooleana-1,9-dien-28-oate, via MAPK pathways, leading to caspase-8 activation [88]. Oleanolic acid derivative, SZC015, activated intrinsic apoptosis, as observed by the up-regulation of caspase-3 and caspase-9, poly (ADP-ribose) polymerase (PARP) cleavage, release of cytochrome c, as well as increase in Bax/Bcl-2 expression ratio [55,87]. Moreover, oleanolic acid and its derivatives also caused autophagy, i.e., formation of autophagic vacuoles, elevated microtubule-associated protein 1 light chain 3α (MAP1LC3A), increased LC3II/LC3I ratio and upregulated the expression of Atg5 and beclin1 in hepatic [50], breast [55] and gastric cancer cells [73,87]. Interestingly, oleanolic acid-triggered autophagy was ROS-dependent as shown by elevated cellular ROS levels, and the effect was abolished if ROS levels were reduced [50,62]. Indeed, increased ROS levels were responsible for the increased anticancer activity in the co-administration of oleanolic acid together with routinely used chemotherapeutic drug (sorafenib) in hepatocellular carcinoma [89]. Mechanistically, oleanolic acid dose- and time-dependently stimulated apoptosis via activation of ROS/apoptosis signal-regulating kinase 1 (ASK1)/p38 MAPK pathway [90]. Oleanolic acid induced the activation of ASK1 mediated by ROS levels which, in turn, phosphorylated p38 MAPK and subsequently activated pro-apoptotic proteins by phosphorylation of Bax, Bim, and Bcl-2 [90].

In a recent study, oleanolic acid modified Warburg-like metabolism, which was induced by high salt-mediated suppression of cytochrome oxidase, caspase cascade as well as the expression of Bax protein. This study suggested that oleanolic acid can cause apoptosis through mitochondrial-related pathways promoting the release of mitochondrial-associated caspases and pro-apoptotic Bax proteins [91]. In addition, oleanolic acid inhibited aerobic glycolysis by inducing pyruvate kinase muscle (PKM)isoforms switch from PKM2 to PKM1 through suppression of phosphorylated mTOR, consequently disrupting Warburg effects in various cancer cells [92].

### 4.3. Oleanolic Acid Mediated Control of TRAIL-Induced Signaling

Tumor necrosis factor (TNF)-related apoptosis-inducing ligand (TRAIL)-mediated signaling is an extensively-studied area of oncology, and has led to developing a sharper understanding of the protein network, which is functionalized to promote apoptosis in cancer cells without affecting non-tumor cells [93,94,95]. TRAIL transduces the signals intracellularly by activating death receptors (DR4 and DR5) situated on the surface of neoplastic cells [96,97]. Structural interaction of TRAIL with its receptors induces clustering of the receptors into high-molecular-weight nano-complexes that facilitates the development of the death-inducing signaling complex (DISC) [95]. DISC formation is necessary to activate caspase-8 [98]. Caspase-8 proteolytically processes a downstream effector caspase-3 [99]. Once caspase-8 is activated, its downstream effector is channelized through receptors and termed as an extrinsic pathway [100]. However, intrinsic pathway is functionalized through entry of truncated Bid (tBid) into mitochondrion. Importantly, tBid promotes the mitochondrial release of cytochrome c [101]. Cytochrome c interacts with pro-caspase-9 and apoptotic protease activating factor to generate the apoptosome [102]. Functionally, active caspase-9 further activates caspase-3 in cancer cells leading to apoptosis activation [103].

TRAIL-induced apoptosis is frequently impaired in cancer cells mainly because of downregulation of DR4 and DR5, inactivation of pro-apoptotic proteins, and overexpression of anti-apoptotic proteins. Different synthetic and natural products have been reported to induce apoptosis in TRAIL-resistant cancer cells. In the following sections, we summarize how oleanolic acid modulated this protein network in cancer cells.

A derivative of oleanolic acid, 3-*O*-acetyloleanolic acid, was found to effectively improve TRAIL-mediated apoptosis in human colon tumor (HCT)-116 cancer cells. Detailed mechanistic insights revealed that 3-*O*-acetyloleanolic acid upregulated DR5 expression in the treated colon cancer cells. Indeed, also markedly enhanced levels of caspase-8 and caspase-3 were noticed in those cells [104].

Synthetically-designed cyanoenone of methyl boswellates (CEMB), a triterpenoid compound, was also reported to exert its anticancer effects via upregulation of DR4. Gene silencing strategy confirmed that CEMB-mediated apoptosis was dramatically reduced in DR4-silenced prostate cancer cells [105].

Another synthetically designed triterpenoid that has entered into clinical trials is methyl-2-cyano-3,12-dioxoolean-1,9-dien-28-oate (CDDO-Me). CDDO-Me transcriptionally upregulated DR5 by an important protein, namely C/EBP homologous protein (CHOP). CHOP binding sites have previously been identified in the promoter region of DR5. Research data showed that CDDO-Me-induced apoptotic effects were significantly impaired in CHOP silenced cancer cells [106]. CDDO-imidazolide potentiated the expression of DR4 and DR5 in acute myeloid leukemia cells, and downregulated decoy receptors (TRAIL-R3/TRAIL-R4) [107].

Overall, accumulating evidence clearly suggests that oleanolic acid plays a significant role in improving TRAIL-mediated cytotoxic activity via upregulation of death receptors, elevation of pro-apoptotic proteins, and inhibition of anti-apoptotic proteins in treated cancer cells.

### 4.4. Oleanolic Acid Inhibited Angiogenesis, Invasion and Metastasis

Oleanolic acid inhibited invasion of tumor cells, angiogenesis and metastasis in several cancer models. For instance, oleanolic acid reduced the rate of lung metastasis in vivo in osteosarcoma [65] and NSCLC [53], as well as inhibited the angiogenesis in colorectal cancer [108].

From an angiogenic perspective, oleanolic acid suppressed the activation of the signal transducer and activator of transcription 3 (STAT3) and sonic hedgehog signaling pathways, which are key pathways in angiogenesis, and downregulated proangiogenic vascular endothelial growth factor A and basic fibroblast growth factor [108].

Pharmacophore modeling study of oleanolic acid was performed to identify novel analogs of oleanolic acid with the aim to improve the inhibition of human breast cancer cells migration, proliferation, and invasion [109]. This study also discovered that Brk/Paxillin/Rac1 pathway plays an essential role in the antimigratory and anti-invasive effects of oleanolic acid and its derivatives [99]. Twelve semi-synthetic analogues of oleanolic acid were tested from which carbamate derivatives, 3-*O*-[*N*-(30-chlorobenzenesulfonyl)-carbamoyl]-oleanolic acid and 3-*O*-[*N*-(50-fluorobenzenesulfonyl)-carbamoyl]-oleanolic acid showed strong and selective anticancer activities against breast cancer cells. The authors concluded that the existence of a sulfonyl-carbamoyl moiety with an optimal bulkiness of electron-deficient phenyl ring was predominantly responsible for the increased antineoplastic activity [98].

### 4.5. Oleanolic Acid Suppressed Multi-Drug Resistance Proteins

Oleanolic acid has antiproliferative and proapoptotic effects in a time- and dose-dependent manner on various multi-drug resistance cancer cell lines in in vitro conditions associated with a down-regulation of apoptosis antagonistic proteins, including Bcl-2, Bcl-xL, and survivin [110]. Oleanolic acid inhibited the effect of the multi-drug resistance-associated protein 1 (MRP1) [111] and was also cytotoxic to the multi-drug resistant erythroleukemic cells overexpressing permeability glycoprotein (P-gp) [112]. Oleanolic acid was able to modulate MRP1 activity without altering its gene expression, but no effects were reported in P-gp activity in a renal cell line expressing constitutively both proteins [111]. Multiple drug resistance is considered to be one of the leading reasons of failure of chemotherapy in cancer patients; therefore, oleanolic acid has potential as a chemotherapeutic agent itself in tumors with high expression of efflux transporters, such as MRP1 or P-gp, or as a co-adjuvant in the chemotherapy of P-gp/MRP1 expressing tumors [111]. However, P-gp and MRPs are also found in non-tumoral tissues. For this reason, it seems to be a prudent approach to use non-toxic inhibitors of efflux transporters, such as oleanolic acid, which is likely to maintain normal physiological function in healthy tissues.

### 4.6. Oleanolic Acid Exerted Synergistic Activity with Chemotherapeutic Drugs

Combined use of oleanolic acid and 5-fluorouracil synergistically potentiated the cytotoxicity on pancreatic cancer cells as well as acted in pro-apoptotic fashion [61]. In a similar manner, oleanolic acid could be combined with other chemotherapeutic agents.

### 4.7. Oleanolic Acid Displayed Radiosensitizing Effects

Oleanolic acid increased the radiosensivity of tumor cells by inhibiting the synthesis of cellular glutathione (GSH) with concurrent inhibition of γ-glutamylcysteine synthetase (γ-GCS), a key enzyme in GSH synthesis [113].

## 5. Increased Selective Toxicity of Novel Oleanolic Acid Derivatives

Importantly, novel oleanolic acid derivatives have been recently synthesized and tested against various human cancer cell lines, and several of those compounds had cytotoxic activity against cancer cells in the low micromolar range (*EC*_50_ = 3–6 µM) and cytotoxicity against non-cancer cells in high micromolar range (*EC*_50_ > 120 µM) providing the conditions for selective toxicity [114,115]. Various oleanolic acid derivatives, including lactams, ketones, oximes, and nitriles, were tested in different cancer cell lines with evidence that lactams and oximes exerted the most cytotoxicity effects [114]. The introduction of acetyl groups at positions C-2 and C-3 and the existence of (2β,3β)-configured centers appears to be necessary for the cytotoxic effects and to obtain the best selectivity between cancer cells and normal mouse fibroblast cells [115]. These results show rationale to employ oleanolic acid and its derivatives as lead compounds in anticancer drug discovery. In addition, novel drug design forms, such as solid inclusion complexes of oleanolic acid with amino-appended β-cyclodextrins [116], multivesicular liposomes for oleanolic acid [117], or oleanolic acid-loaded PEGylated polylactic acid, and polylactic-co-glycolic acid nanoparticles [118], improved oleanolic acid bioavailability and, thus, increased its anticancer potency.

## 6. Perspectives on Using Oleanolic Acid as an Adjuvant of Cancer Treatment

Natural product chemistry has undergone substantial broadening and many of the phytochemicals have entered various phases of clinical trials. Oleanolic acid has attracted considerable attention because of its cancer inhibitory roles via regulation of different signaling cascades (summarized in Table 2). However, the existing information related to how oleanolic acid modulates different proteins of different intracellular signaling networks is still incompletely studied. We still need to intensely study cell-type specific and context-dependent effects exerted by oleanolic acid on different proteins either to inhibit cancer progression, or to induce apoptosis. Importantly, how oleanolic acid re-balances pro-apoptotic and anti-apoptotic protein stoichiometric ratios needs detailed future research. The most consistent pathway reported involves the induction of autophagy by oleanolic acid via suppressing mTOR through inhibition of the PI3K/AKT and ERK/p38 MAPK signaling pathways and activation of the AMPK signaling pathway. Certain hints have also emerged highlighting how oleanolic acid differentially modulates oncogenic and tumor suppressor miRNAs. Nevertheless, we have to develop a better understanding of the miRNAs which are activated or inhibited in oleanolic acid-treated cancer cells. Different signaling cascades particularly, Wnt, sonic hedgehog, TGF, Notch, and Janus kinase-STAT, have to be comprehensively investigated in different cancers. These aspects will provide a better comprehension regarding to molecular targets underlying anticancer effect of oleanolic acid. Several studies have also documented that plant extracts belonging to different families and containing oleanolic acid are effective against many cancer cell lines. However, up to date no comparisons between the extract and the isolated compounds have been designed in order to determine additive or synergistic effects of the extracts with respect to the individual compounds.

## 7. Conclusions

Oleanolic acid has the potential to be employed in clinical practice in adjuvant anticancer treatment because: (i) it has direct anticancer activity and can act synergistically with chemotherapeutic drugs; (ii) it inhibits efflux transporters, thereby increasing the intracellular concentration of chemotherapeutic drug; (iii) it exhibits a radiosensitizing effect for irradiation-induced cell death, thereby increasing the efficacy of radiation therapy; and (iv) it has relatively low toxicity and does not have any adverse effects when combined with chemotherapeutic agents. Additionally, anticancer properties of oleanolic acid was in the low micromolar range, which corresponds to the physiologically relevant values obtainable upon oleanolic acid-enriched diet, thereby offering the potential to use oleanolic acid as a vital part of healthy diet. The main limitation of the potential therapeutic use of oleanolic acid is the absence of clinical trials which are necessary to confirm the promising data about its chemoprotective and anticancer effects. In addition, similar to other phenolic compounds, the poor bioavailability limits its potential effects and makes it necessary to find new formulations that increase its bioavailability and effectiveness.

## Figures and Tables

**Table 1:** Main sources of oleanolic acid.

Fruits	Analyzed Part	Oleanolic Acid Concentration	Method	Reference
Apples	Pomace	16 µg/g·dm	HPLC-DAD	[44]
	Skin	28 µg/g·dm		
Pomegranate	Sarocarp	nd	HPLC-DAD	[45]
	Peel	26.96 ± 0.93 µg/g dw		
	Seed	1.12 ± 0.09 µg/g dw		
Lemon	Sarocarp	nd		
	Peel	0.62 ± 0.01 µg/g dw		
Mandarin	Sarocarp	nd		
	Peel	1.05 ± 0.04 µg/g dw		
Bilberries	Whole fruit	1679.2–2029.6 µg/g dw	GC-MS-FID	[25]
Pears	Skin	164.3–3066.6 µg/g fw	HPLC-PAD	[46]
	Pulp	34.0–156.0 µg/g fw		
Grapes	Peel	176.2 µg/g dw	HPLC-FD	[47]
Persimmon	Peel	367.7 µg/g dw		
	Flesh	17.2 µg/g dw		
Jujube	Pulp	360 ± 10.7 µg/g dw	UHPLC-MS/MS	[30]
Olives	Skin	3094–4356 µg/g fw	HPLC-DAD	[48]
	Pulp	27–29 µg/g fw		
	Seed	nd		

dm, dry matter; dw, dry weight; fw, fresh weight; nd, not detected.

**Table 2:** Main anti-cancer effects of oleanolic acid.

Inhibition of tumor initiation and promotion	Cell cycle arrests [52,60,71]
	Inhibition of PI3K/Akt/mTOR/NF-κB signaling pathway [55]
	Inhibition of mitogen-activated protein kinase (ERK/JNK/p38 MAPK) signaling pathways [55,68,73]
	Activation of AMPK signaling pathway [74]
	Inhibition of TGF-β by binding to its receptors [76,78]
	Inhibition of topoisomerase I and IIα proteins [63]
Apoptosis induction	Elevation cytochrome c release [51,55]
	Decrease anti-apoptotic Bcl-2 proteins [51,55,87,90]
	Increase pro-apoptotic Bax proteins [55,87,90]
	Up-regulation of caspases [21,51,53,61,62,66,86,91]
	Poly (ADP-ribose) polymerase (PARP) cleavage [55,56,57,58,59,60,61,62,63,64,65,66]
	Induction of autophagy [50,55,73,87]
	Disruption of Warburg metabolism [91,92]
	Induction of TRAIL mediated apoptosis [104,105,107]
	Inhibition of the multi-drug resistance associated proteins effects [110,111,112]
Metastasis inhibition	Suppression of STAT3 and sonic hedgehog signaling pathways [67,108]
	Downregulation of proangiogenic vascular endothelial growth factor A and basic fibroblast growth factor [108]
